# Sensing-actuating integrated asymmetric multilayer hydrogel muscle for soft robotics

**DOI:** 10.1038/s41378-025-00884-9

**Published:** 2025-03-04

**Authors:** Yexi Zhou, Yu Zhao, Dazhe Zhao, Xiao Guan, Kaijun Zhang, Yucong Pi, Junwen Zhong

**Affiliations:** Department of Electromechanical Engineering and Centre for Artificial Intelligence and Robotics, University of Macau, 999078 Macau SAR, China

**Keywords:** Electronic properties and materials, Electrical and electronic engineering

## Abstract

Achieving autonomously responding to external stimuli and providing real-time feedback on their motion state are key challenges in soft robotics. Herein, we propose an asymmetric three-layer hydrogel muscle with integrated sensing and actuating performances. The actuating layer, made of p(NIPAm-HEMA), features an open pore structure, enabling it to achieve 58% volume shrinkage in just 8 s. The customizable heater allows for efficient programmable deformation of the actuating layer. A strain-responsive hydrogel layer, with a linear response of up to 50% strain, is designed to sense the deformation process. Leveraging these actuating and sensing capabilities, we develop an integrated hydrogel muscle that can recognize lifted objects with various weights or grasped objects of different sizes. Furthermore, we demonstrate a self-crawling robot to showcase the application potential of the hydrogel muscle for soft robots working in aquatic environments. This robot, featuring a modular distributed sensing and actuating layer, can autonomously move forward under closed-loop control based on self-detected resistance signals. The strategy of modular distributed stimuli-responsive sensing and actuating materials offers unprecedented capabilities for creating smart and multifunctional soft robotics.

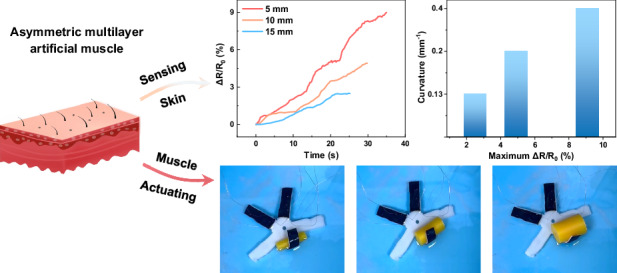

## Introduction

Soft robotics offer significant benefits in terms of safety and adaptability, making them highly versatile for biomedicine, prosthetic, and perception technologies^1–4^. Stimulation-responsive polymers are particularly promising for creating soft robots due to their customizable composition and structure, exhibiting changes in electrical signals, or undergoing substantial deformation when exposed to external stimuli^5–7^. A key challenge that remains is the development of soft robotics capable of autonomously responding to external stimuli and providing real-time feedback on their motion state^8,9^. These robotics have the potential to monitor various properties of the objects they interact with, such as roughness^10^, temperature^11^, weight^12^, and surface components^13^. Recent advancements in soft sensor and actuator technology, including those based on static electricity^14–16^, piezoelectricity^17–19^, piezoresistivity^20^, and electromagnetic^21–24^ have greatly contributed to the development of self-sensing robotics.

Hydrogels have rapidly advanced in stimuli-responsive polymer-based soft robotics due to their ability to respond to mechanical strain^25–27^, temperature^28,29^, pH, electricity, and solvents, depending on their composition^30–32^. Thermal-induced deformation is a convenient method for fabricating hydrogel actuators^33–35^. One common approach to achieving self-sensing functionality is the chemical integration of sensing and actuation components into a single hydrogel^36–38^. This is often done by introducing conductive materials like carbon nanotubes, graphene, and black conducting polymers into thermally responsive poly(N-isopropylacrylamide) (PNIPAm) hydrogels, creating dual-functional conductive hydrogels. As the PNIPAm hydrogel networks shrink when the temperature exceeds its lower critical solution temperature (LCST), the number of conductive paths increases, leading to decreased resistance. Another effective method for producing shape deformation and self-sensing is the fabrication of multilayer structures^39–41^. When the PNIPAm layer shrinks under external stimuli, other layers bend passively towards it due to the volume mismatch. This bending reduces the number of conductive paths in the passive layer, thereby increasing the resistance. Multilayer structures offer high design flexibility and more accurate sensing performance. By utilizing self-sensing functionality, the shape transformation process can be detected in real-time, allowing for more efficient control of the actuation process. However, the use of photothermal effects to drive the actuator in the above-mentioned studies^36–39^ often results in low energy conversion efficiency and weak controllability, limiting the potential applications of self-sensing hydrogel muscles.

In this work, we develop a novel hydrogel muscle with integrated shape deformation and sensing functionality. Inspired by the collaboration between skin and muscles, we fabricate an asymmetric three-layer hydrogel muscle that meets the sensing, actuating, and real-time feedback requirements. The sensing layer consists of p(AAm-AA) hydrogel doped with carbon nanotubes (CNT) and liquid metal (LM). These conductive fillers enable the hydrogel to maintain a conductivity of approximately 0.58 S/m in water for over 30 days. Its resistance exhibits a linear response to low-strain deformation and remains consistent across different drawing frequencies. The actuating layer is made of p(NIPAm-HEMA) hydrogel, which can achieve 58% volume shrinkage in 8 s when exposed to 45 °C water and return to its initial volume in 60 s after being put in room temperature water. Heaters made of serpentine copper wires are embedded between the hydrogel layers, allowing the muscle to rapidly change to any arbitrary shape and size. As a result, we develop open-loop controlled hydrogel muscles that can recognize the weight or size of unknown objects based on deformation time and resistance changes. Additionally, we create a closed-loop controlled self-crawling robot to demonstrate the hydrogel muscle’s potential in soft robotics. This inchworm-like robot can autonomously crawl forward, controlled by self-sensed resistance during movement. This work highlights the significant potential of self-sensing hydrogel muscles in fully soft robotics.

## Results and discussion

### Working principle and design strategy

**Fig. 1:**
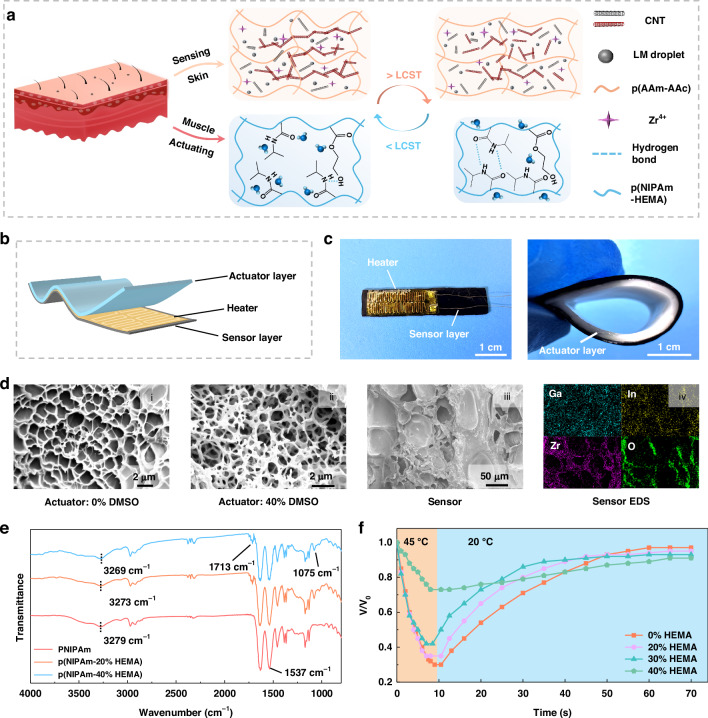
**Working principle and design strategy**. **a** Schematic diagram of the working principle of the sensing and actuating layers, in which LCST means the lower critical solution temperature. **b** Schematic diagram indicating the structure design of the three-layer hydrogel muscle. **c** Images of the fabricating process and fabricated hydrogel muscle. **d** Cross-section SEM images of the actuating and sensing layer, and EDS images of the sensing layer. **e** FTIR image of the actuating layer with different HEMA content. **f** Deswelling (45 °C) and swelling (20 °C) process of the actuating layer with different HEMA content

Figure 1a demonstrates the working principle of the hydrogel muscle. Inspired by the skin and muscle structure of mammals, a hydrogel muscle composite with integrated sensing and actuating capabilities is fabricated. The sensor layer is formed through in situ polymerization of AAm and AA within aqueous dispersions of CNT and LM. It is immersed in a 0.1 M ZrClO_2_ solution for 2 h to impart anti-swelling properties. The actuator layer is produced by polymerizing NIPAm and 2-hydroxyethyl methacrylate (HEMA) in a mixed solvent of dimethyl sulfoxide (DMSO) and water at 0 °C (Figs. S1 and S2). The actuating layer becomes more hydrophobic when the temperature exceeds its LCST, leading to water discharge and volume shrinkage. Consequently, the hydrogel muscle bends towards the actuator side as the sensor layer remains non-contractive. This bending reduces the conductive path in the sensing layer, increasing its resistance. Therefore, the bending process can be monitored by measuring the resistance of the sensing layer. To achieve precise control over the bending process of the hydrogel muscle, a heater is integrated between the two hydrogel layers (Fig. 1b). The serpentine copper heater, encapsulated in PI, is directly affixed to the sensing layer hydrogel. The sensing and actuating hydrogel layers are bonded by octadecene-diluted n-butyl isocyanate glue (Fig. 1c). This technique results in a multilayer structure that offers excellent flexibility and ensures a seamless interface between each layer.

Figure 1d(i) and (ii) present the cross-sectional SEM images of the actuating layer synthesized with different DMSO fractions. The actuating layer fabricated using a mixed solvent with a 40% mole fraction of DMSO exhibits an open pore structure, which significantly enhances the kinetics of deswelling and swelling during thermal transitions. This open pore structure is further facilitated by the low-temperature polymerization environment (Fig. S3). The reason is that the chains of hydrogels collapse into spheres during the reaction under this condition. Due to the high cross-linking degree of the microsphere itself and the low cross-linking degree of the surrounding microsphere, the structure of an open hole is caused^35,42^. The cross-section image in Fig. 1d(iii) reveals a dense structure in the sensing layer, ensuring its anti-swelling properties. The EDS images confirm the uniform dispersion of LM and Zr^4+^ within the hydrogel matrix (Fig. 1d(iv)).

To speed up the response and enhance the mechanical strength of the actuator layer, HEMA is copolymerized with NIPAm. Figure 1e shows the FTIR spectra of the hydrogel with different HEMA content. The new peaks in 1713 and 1075 cm^-1^ are associated with C=O and C−O stretching vibration, proving the successful copolymerization of HEMA. The characteristic peak at 3279 cm^-1^ is associated with the N−H stretching in NIPAm. With the increase of HEMA content in the hydrogel, this peak gradually shifts to the lower wave number due to the intramolecular hydrogen bonds between the hydroxyl groups in HEMA and the amide groups in NIPAm. The LCST of the actuator layer decreases as the HEMA content increases from 0% to 30%^43–45^, resulting in an accelerated deswelling rate at a high temperature. Conversely, the swelling rate increases, and deswelling volume reduces from 70% to 58% within this HEMA content range due to the hydrophilicity of HEMA (Fig. 1f and Fig. S4, Video S1). However, at 40% HEMA content, hydrophilicity predominates the working performance. As evidenced by the SEM image (Fig. S5), the hydrogel shows a dense and close pore structure when HEMA content is 40%, leading to decreased deswelling speed and volume shrinkage. The mechanical strength of the actuating layer exhibits a similar trend, with the maximum stress increasing from 7 kPa to 17 kPa as the HEMA ratio rises from 0% to 30% (Fig. S6) but decreases when the content exceeds 40%. Toughness is evaluated by applying a 1500 g load on hydrogels with 30% and 40% HEMA content. The actuating layer with 40% HEMA fractures, whereas the one with 30% HEMA remains intact (Fig. S7). Considering both response time and mechanical strength, the actuator layer with a 30% HEMA weight ratio is selected for further testing.

### Thermo-responsive actuation behavior optimization and characterization

**Fig. 2:**
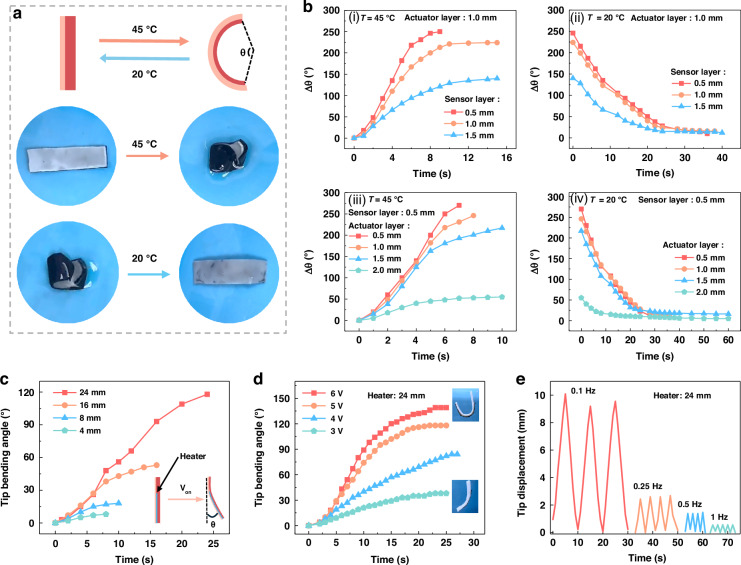
**Thermo-responsive actuation behavior optimization and characterization**. **a** Schematic diagram and images of the thermal-responsive bending (45 °C) and recovery (20 °C) process of the hydrogel muscle. **b** Influence of the thickness of the sensing and actuating layer on the bending and recovery process. **c** The bending process of hydrogel muscle with different heater lengths. **d** The bending process of hydrogel muscle with a 24 mm heater under different driving voltages. **e** The tip displacement of hydrogel muscle with a 24 mm heater at different actuating frequencies

The deformation process of a bilayer hydrogel composite is depicted in Fig. 2a. Upon exceeding the LCST of the actuating layer, the hydrogel composite bends towards the actuating layer and reverts to its planar state when immersed in water below the LCST (Video S2). The sensing layer, functioning as a passive component, impedes hydrogel deformation, and the actuating layer directly induces the deformation force, which means the thickness of both layers critically influences the deformation performance. The bending angle of a hydrogel composite, with 1 cm in width and 4 cm in length, is utilized to evaluate the actuation performance across various thicknesses of the actuating and sensing layers. As illustrated in Fig. 2b(i) and (ii), with a 1 mm actuating layer, the bending speed diminishes as the sensor layer thickness increases, and the bending angle decreases from 250° to 140° when immersed in 45 °C water. The recovery speed in 20 °C water also declines as the sensor layer thickness increases from 0.5 mm to 1.5 mm. To assess the impact of actuator layer thickness, the sensing layer is maintained at 0.5 mm (Fig. 2b(iii) and (iv)). The bending and recovery speeds, along with the final bending angle, exhibit minimal variation when the actuator layer is thinner than 1.5 mm. However, at a thickness of 2 mm, both the bending speed and angle are reduced due to the decreased volume mismatch between the two layers after deswelling. Based on these findings and considering the mechanical strength of the hydrogel actuator, a 0.5 mm sensing layer, and a 1 mm actuating layer are selected for subsequent experiments.

The heater length (Fig. S8) and the applied voltage have an important influence on the bending angle and radius of the actuator. As shown in Fig. 2c, the tip bending angle increases from 30° to 120° when the heater length increases from 4 mm to 24 mm. The maximum bending angle is from 38° to 139° when the voltage is from 3 V to 6 V. It rapidly increases from 3 V to 5 V, but the bending speed and maximum bending angle show little increase after exceeding 5 V (Fig. 2d and Video S3**)**. According to the above results, the bending process can be precisely controlled by the heater length and voltage duty cycle. Figure 2e shows the hydrogel muscle vibrates at 0.1 to 1 Hz by controlling the voltage duty cycle. The maximum tip displacement at 0.1 Hz is about 10 mm and greatly decreases with increased frequencies. The maximum generated force of the actuator is about 42 mN when the heater length is 24 mm and decreases to 5 mN with a 4 mm heater length, as shown in Fig. S9.

### Working performance of the sensing layer

**Fig. 3:**
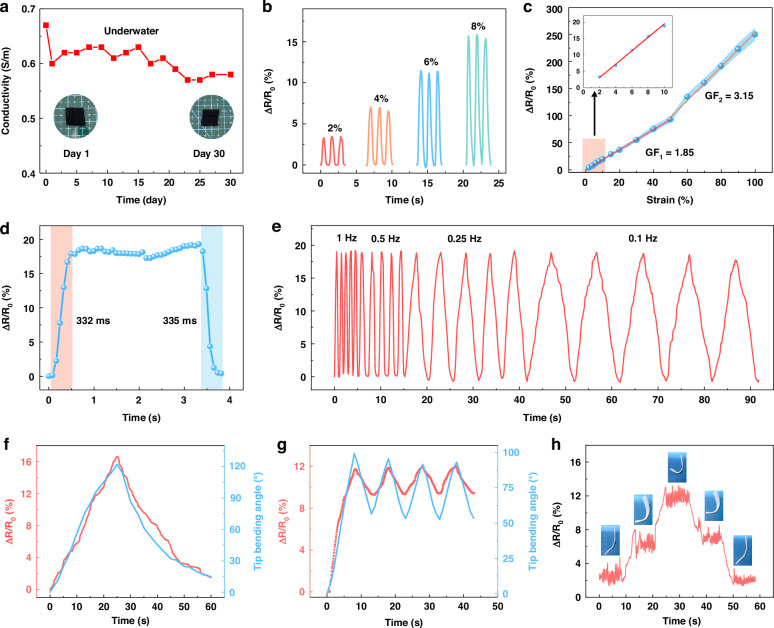
**The working performances of the sensing layer**. **a** Conductivity of the sensing layer after immersing in water for the duration test. **b** Resistance changes of the sensing layer at 2%, 4%, 6%, and 8% strain. **c** Relative resistance changes in the 0-100% strain and the corresponding gauge factor (GF). **d** Response time and release time of the sensing layer at 10% strain. **e** Resistance changes of sensing layer at 10% strain under 0.1–1 Hz. **f** The resistance changes of the hydrogel muscle during the full bending and recovery process. **g** The resistance changes of hydrogel muscle at 0.1 Hz driving frequency. **h** Relative resistance changes of hydrogel muscle maintaining different bending angles

The introduction of CNT and LM provides the sensing layer with good conductivity, which makes it capable of serving as a strain sensor (Fig. S10). Because the electrical conductivity is mainly supplied by electron migration rather than ion mobility, coupled with its excellent anti-swelling properties, the conductivity of the sensing layer shows little reduction after soaking in water for more than 30 days (Fig. 3a). The resistance variation ratios (ΔR/R_0_) of the hydrogel under different strains are studied to evaluate the working performance of the sensing layer. As shown in Fig. 3b, the hydrogel shows repeatable resistance change during loading and recovery at strains from 2% to 8%. Resistance change shows a linear relationship with the stretching ratio. The gauge factor (GF) is employed to assess the sensitivity of the sensing layer. The GF value is 1.85 in the low-strain range of 0-50% and increases to 3.15 in 60–100% (Fig. 3c). This is caused by the rapid increase of discontinuity in the conductive network under large deformation. The introduction of LM increases sensitivity by about 2.4 times (Fig. S11). An accurate measurement of the response time during the stretching and release process is shown in Fig. 3d. Under a sudden loading and unloading of a 10% strain process, the response and recovery time are 332 ms and 334 ms, respectively. The excellent responsiveness ability enables the sensing layer to monitor different strain frequencies. The hydrogel shows a consistent resistance variation at 0.1 to 1 Hz under a given 10% strain process (Fig. 3e).

The maximum temperature during the electrothermal actuation process is about 34.4 °C (Fig. S12), which has little effect on the resistance of the sensing layer. Therefore, the resistance change during the actuation process is mainly caused by the deformation of the hydrogel. Figure 3f illustrates the resistance change during a full actuation and recovery process. The maximum resistance change is about 16% with a tip bending angle of about 120^o^. At a 0.1 Hz vibration process, the resistance variation ratio changes from 11.7% to 9.3%. The resistance curve and tip displacement from 98° to 73° show the same trend (Fig. 3g). Moreover, the sensing layer accurately records the resistances at the given bending angles (Fig. 3h). The sensing layer shows a stable resistance change of about 2.5%, 6.2%, 11.5% when the actuating layer bends to and maintains at about 15°, 60°, 95°. These results indicate the hydrogel muscle has reproducible and reliable sensing performance, which shows the potential for self-sensing applications.

### Self-sensing behaviors of the hydrogel muscle

**Fig. 4:**
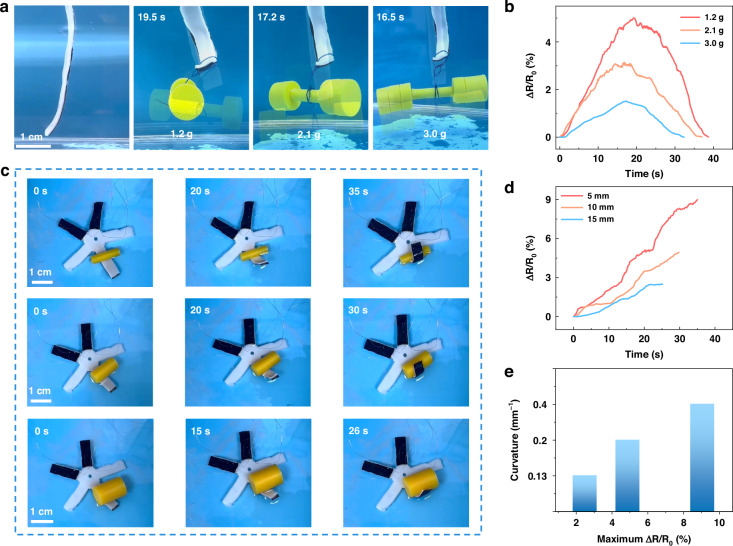
**Self-sensing behaviors of the hydrogel muscle.** **a** Images of the hydrogel muscle lifting loads of different weights. **b** Relative resistance changes of the sensing layer during the lifting process. **c** Images of a starfish-like robot wrapping objects with different sizes. **d** Relative resistance changes of the sensing layer when wrapping different-sized objects. **e** The correlation between resistance changes and curvatures of objects

Proprioception is further demonstrated through real-time monitoring of actuation behaviors, emulating the self-sensing capabilities of living organisms. We illustrate the kinesthetic lifting of various loads using a hanging hydrogel muscle. The power and sensing system is shown in Fig. S13. As depicted in Fig. 4a, the maximum lifting time decreases from 19.5 s to 16.4 s as the load weight increases from 1.2 g to 3 g. The final bending angle and recovery speed also vary with the load weight. These regulated motions are effectively monitored in real-time by the sensing layer. The maximum resistance change during the lifting of a 1.2 g load is about 5% at 19.5 s, gradually returning to the initial value at 38.5 s (Fig. 4b). The lifting processes of 2.1 g and 3.0 g loads, with smaller deformation and reduced actuation time, demonstrate resistance changes of about 3.1% and 1.5%, respectively. The resistance change curve trends are consistent with the bending and recovery processes recorded in Video S4. To demonstrate the utility of the integrated sensing and actuating functionalities, a starfish-shaped soft robot is fabricated, incorporating three sensing hydrogels into the robot’s arms. Starting from a straight inactive state, the arms bend upon activation and wrap around cylindrical tubes of varying sizes (Fig. 4c). As shown in Fig. S14, the hydrogel muscle demonstrates low sensitivity to pressure caused by weights. The recorded resistance values represent the process of perceiving and grasping tubes of different diameters (5 mm, 10 mm, and 15 mm; Video S5). Specifically, a 9% resistance increase occurs when the arm fully wraps around the 5 mm tube in 35 s, whereas a 2.5% resistance increase is observed over a shorter period when grasping the 15 mm tube, reflecting the smaller required bending deformation (Fig. 4d). Thus, both the magnitude and duration of resistance change during grasping provide the potential for shape recognition of unknown objects through the proprioceptive grasping strategy (Fig. 4e). Additionally, beyond sensing performance, the starfish-like robot shows the potential for movement or grasping and then lifting objects through the actuation of different arms (Fig. S15, Fig. S16, and Video S6).

### Demonstration of self-crawling robot

**Fig. 5:**
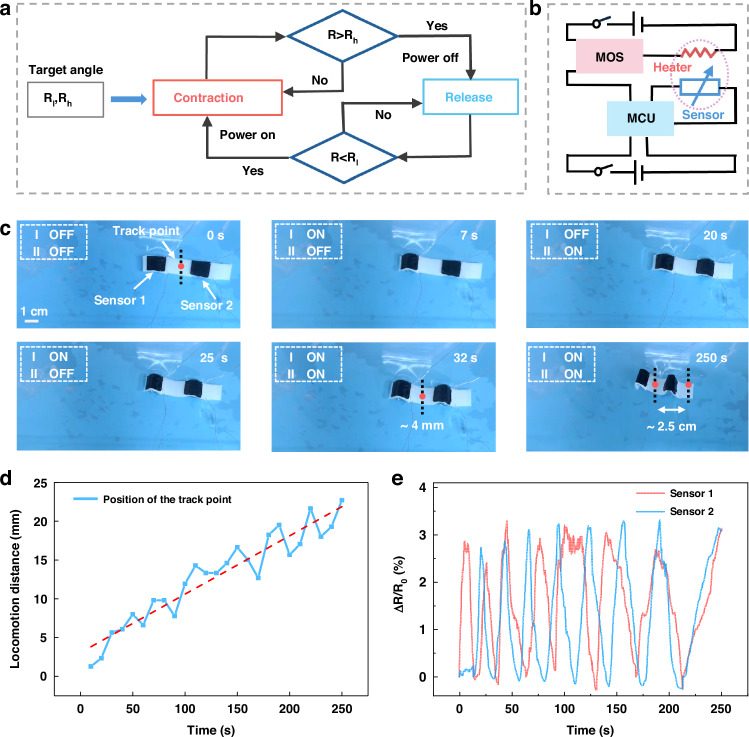
**Demonstration of self-crawling robot**. **a** Workflow chart of the self-crawling robot. **b** Schematic diagram and connection method between hydrogel muscle and control circuit. **c** The sequential steps during the crawling stride under closed-loop control. **d** Plots of self-crawling robot locomotion displacement during 7.5 times of crawling. **e** The relative resistance values of the sensing layer during the clawing process

As a key demonstration of the hydrogel muscle’s unification of sensory and actuation functions, we develop a closed-loop controlled self-crawling robot to mimic a biological neuromuscular system. Benefiting from the module fabricating process, the actuating layer can achieve programmable deformation by placing the heater at different parts (Fig. S17). A control circuit is designed to regulate the bending and recovery deformation of the actuating layer. Specifically, the microcontroller unit (MCU) decides if the power is connected or disconnected to the heater according to the resistance values recorded by the sensing layer. When the resistance of the sensing layer is lower than the low threshold (*R*_l_), the pin of the MOS connected to the MCU is set high to power the heater, and the hydrogel muscle bends downward. As the bending angle increases, the resistance gradually becomes higher than the high threshold (*R*_h_), and the pin will be set low to allow the actuating layer to recover (Fig. 5a, b). The detailed control system and connection method are shown in Figs. S18 and S19. An inchworm-like robot fabricated with a 1 × 5 cm actuating layer, two 1 × 1 cm sensing layers, and two 1 × 1 cm heaters can crawl forward under the control of self-sensed resistance values. The crawling process is divided into four different processes. First, the front heater is activated to the set angle. Then, the back heater is activated, and the front part starts to recover. Due to the increased friction force, the backward motion caused by the front recovery is effectively blocked. Thereafter, the back part gradually recovers, and the front heater is activated during this period. Finally, the two heaters are activated to achieve the maximum moving distance. The first crawling cycle takes about 32 s, and the robot advances about 4 mm (Fig. 5c). Since the recovery speed of the driving layer varies each time during the crawling process, the advanced distance is about 23 mm after autonomous crawling for 250 s, with each actuating module working for 7.5 times (Fig. 5d and Video S7). The moving speed shows a linearity of about 0.075 mm/s. The Δ*R*/*R* signals of the front (sensor 1) and back sensor (sensor 2) are recorded during the moving process. The maximum resistance variations are about 3% monitored from both sensors (Fig. 5e). The trend in these curves is the same as the bending processes of the two actuating modules (Video S7), which confirms that actuation and real-time sensory feedback can be perfectly integrated.

## Conclusions

In summary, we develop an entirely soft hydrogel muscle with integrated sensing functionality. This innovation is achieved by combining a thermally responsive p(NIPAm-HEMA) hydrogel for actuation, a serpentine copper wire heater for control, and a strain-responsive hydrogel sensor for detecting deformation. The actuator layer, with its open pore structure, exhibits rapid deswelling and swelling, achieving up to 58% volume change at 45 °C. The electrothermal-induced stimuli enable the hydrogel muscle to efficiently perform highly programmable deformations. The incorporation of CNT and LM conductive particles endows the sensing hydrogel with excellent sensing properties, allowing it to respond quickly to tensile deformation within the range of 1–50%, fixed-length stretching at 0.1–1 Hz frequency, or bending at various angles. Leveraging these capabilities, the integrated hydrogel muscle can identify the weight of lifted objects, or the size of grasped objects based on real-time resistance variations. Additionally, the hydrogel muscle demonstrates potential applications in soft robots operating in aquatic environments. A robot, modularly composed of sensing, actuating, and heating layers, can autonomously move forward under closed-loop control guided by self-detected resistance signals. Future challenges include (i) optimizing the actuating layer to shorten the response time, (ii) developing a more stretchable heating layer for better integration with hydrogels, (iii) creating robots capable of moving in multiple directions, and (iv) incorporating functional material modules for weight sensing and more application scenarios.

### Experimental section

#### Materials

N-isopropylacrylamide (NIPAm), Acrylic acid (AA), 2-hydroxyethyl methacrylate (HEMA), acrylamide (AAm), dimethyl sulfoxide (DMSO), zirconium chlorooxidized octahydrate (ZrOCl2·8H2O), N, N’-methylenebis(acrylamide) (BIS), 2-hydroxy-2-methyl-1-phenyl-1-propanone (I1173), Tween 80 and 1-octadecene were purchased from Aladdin Reagent Co., Ltd. in China. Multi-walled carbon nanotubes (MWCNT) were supplied by XFNANO Material Tech Co., Ltd. in China. Gallium indium alloys (EGaIn, 75 wt% Ga, and 25 wt% In) were purchased from Zeyang Metal Co., Ltd. All chemicals were used as received without further modification.

#### Preparation of the sensor layer hydrogel

Initially, 50 mg of Tween 80 was dissolved in 6 mL of deionized water using magnetic stirring at room temperature for 5 min. Subsequently, 50 mg of CNT and 100 mg of EGaIn were added to the solution, followed by sonicating in the ice bath for 5 min to achieve uniform dispersion of the conductive particles. Thereafter, 1.5 g of AAm, 0.5 g of AA, and 5 mg of BIS were added into the suspension, which was then stirred at room temperature for an additional 5 min. The suspension was then subjected to sonication for 1 min and poured into silicon molds (thicknesses: 0.5 mm, 1 mm, and 1.5 mm). Electrodes for resistance measurement were directly inserted into the unreacted suspension. The molds were left at room temperature for 2 h to stabilize the polymerization process. Finally, the obtained hydrogel was immersed in 0.1 M ZrOCl_2_ for 2 h to form the coordination bond between COO^-^ and Zr^4+^ to enable the sensor layer anti-swelling^26,27^.

#### Preparation of the actuator layer hydrogel

Typically, 2.4 g of NIPAm, 0.72 g of HEMA, 30 μL of I1173, and 90 mg of BIS were added to a mixed solvent consisting of 4.46 g of DMSO and 1.54 g of deionized water, and the mixture was stirred at room temperature for 10 min^37,42,46^. The resulting solution was then poured into silicon molds (thicknesses: 0.5 mm, 1 mm, 1.5 mm, and 2 mm). The molds were stored at −30 °C for 30 min and subsequently polymerized under 365 nm UV light in an ice bath for 160 s. The polymerized hydrogel was soaked in deionized water for 12 h to remove the DMSO and unreacted chemicals.

#### Fabrication of the hydrogel muscle

First, serpentine copper heating traces (thickness: 20 μm, width: 150 μm) of varying lengths were prepared by laser cutting (LPKF ProtoLaser U4). The copper wires were soldered to the heating trace, and the heating trace was subsequently encapsulated with 5 μm thick PI films. The fabricated heater was directly attached to the sensor layer. Subsequently, octyl cyanoacrylate (Jingu Adhesive) dispersed in 1-octadecene (1:4) was brushed onto the sensor layer. Finally, the actuator layer hydrogel was attached to the sensor layer and pressed for 5 min^47^.

### Characterization

The FTIR spectra were measured by IRSpirit-X (Shimadzu Co., Ltd) employed in ATR mode, with a scanning range of 4000-500 cm^-1^. The resistance of the sensor layer was measured by Keithley 2400 (Tektronix Co. Ltd). The generated force was measured by Mark-10 Series 7 (MARK-10). The cross-section image and EDS were obtained by scanning electron microscopy (Sigma FESEM, Zeiss Co., Germany). The strain-stress test was measured by a tensile machine (8010, CTM).

## Supplementary information

Supplementary Materials

Video S1

Video S2

Video S3

Video S4

Video S5

Video S6

Video S7
