# Supplementary material for: Sensing-actuating integrated asymmetric multilayer hydrogel muscle for soft robotics
Source: Microsyst Nanoeng. 2025 Mar 4;11:40. doi: 10.1038/s41378-025-00884-9 (PMC11876583; doi:10.1038/s41378-025-00884-9)
Supplement: Supplementary file 1 — Supplementary Materials [file 41378_2025_884_MOESM1_ESM.docx]

Supplementary Materials For

**Sensing-actuating integrated asymmetric multilayer hydrogel muscle for soft robotics**

Yexi Zhou^1^, Yu Zhao^1^, Dazhe Zhao^1^, Xiao Guan^1^, Kaijun Zhang^1^, Yucong Pi^1^, Junwen Zhong^1*^

^1^ Department of Electromechanical Engineering and Centre for Artificial Intelligence and Robotics, University of Macau, Macau SAR, 999078, China.

Corresponding author email: [junwenzhong@um.edu.mo](mailto:junwenzhong@um.edu.mo)

**This document includes**

Supplementary Figs. S1 to S19

Supplementary Video captions

**Other supplementary material for this manuscript includes：**

Supplementary Videos S1 to S7


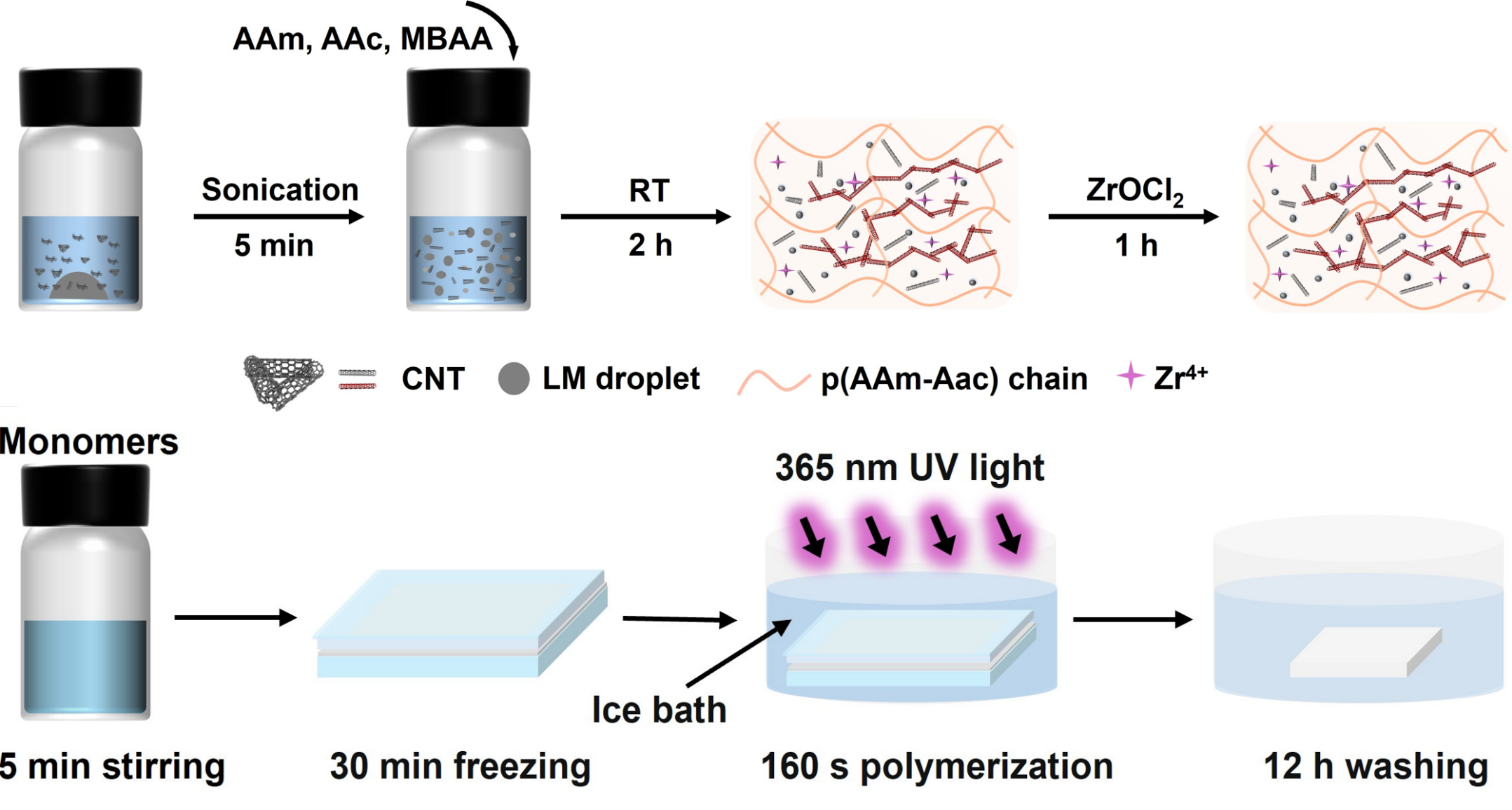


**Fig. S1.** Detailed fabrication process of the sensing and actuating hydrogels.


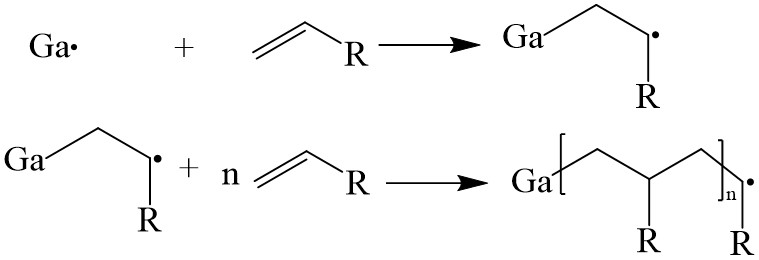


**Fig. S2.** The mechanism of the polymerization process of the sensing layer.


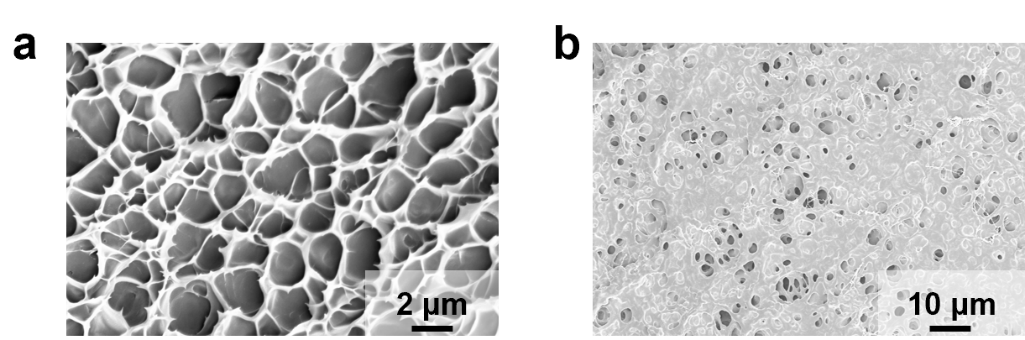


**Fig. S3.** Cross-section SEM images of the actuating layer hydrogel prepared in pure **a** water at 0℃ and **b** in 40% DMSO at room temperature.


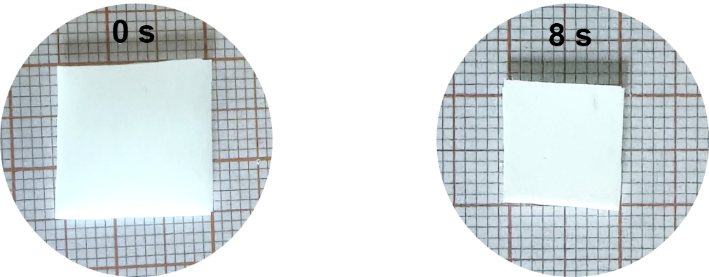


**Fig. S4.** Images of the actuating layer hydrogel before and after volume shrinkage.


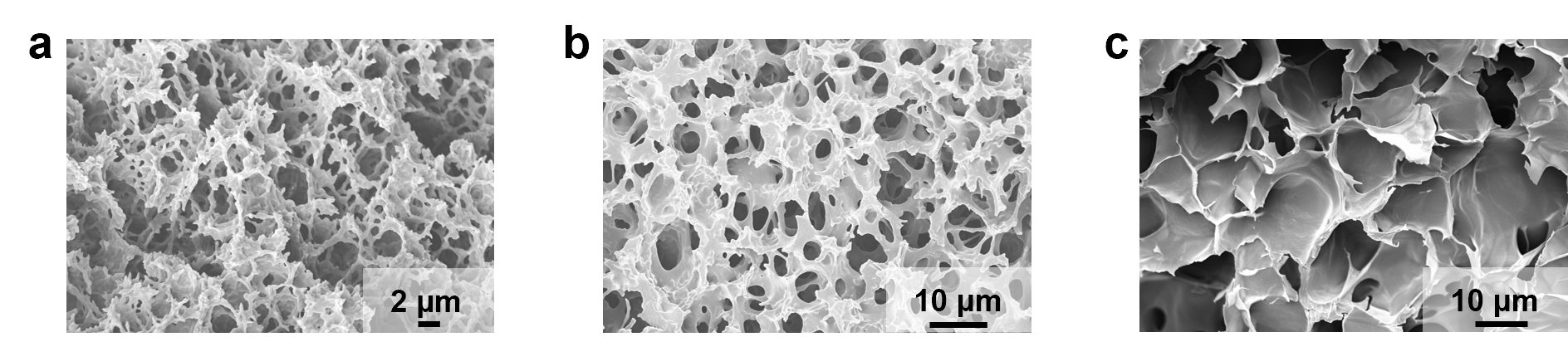


**Fig. S5.** Cross-section SEM images of the actuating layer with **a** 10%, **b** 20%, and **c** 40% HEMA content.


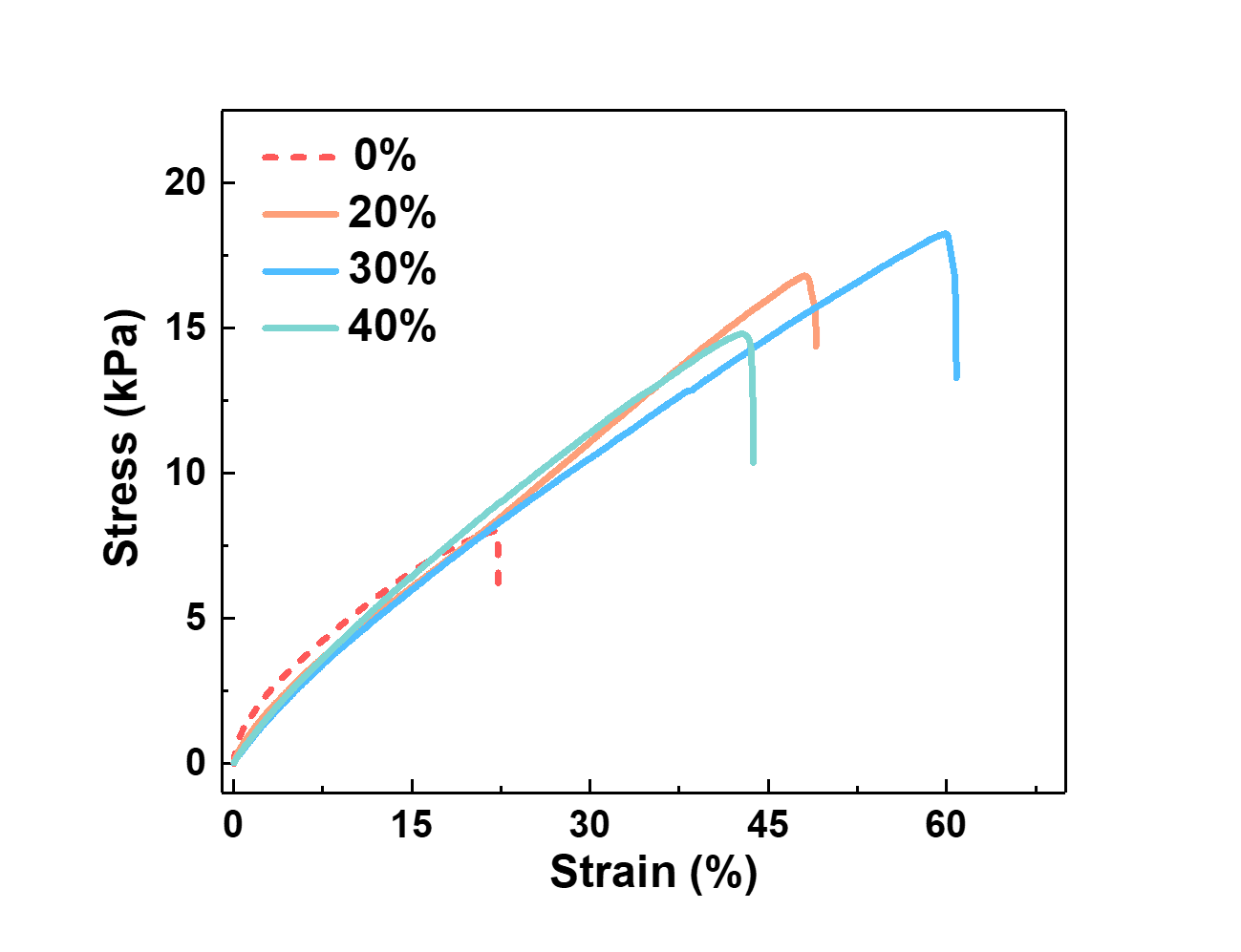


**Fig. S6.** Strain-stress curves of the actuating layer with 0% to 40% HEMA content.


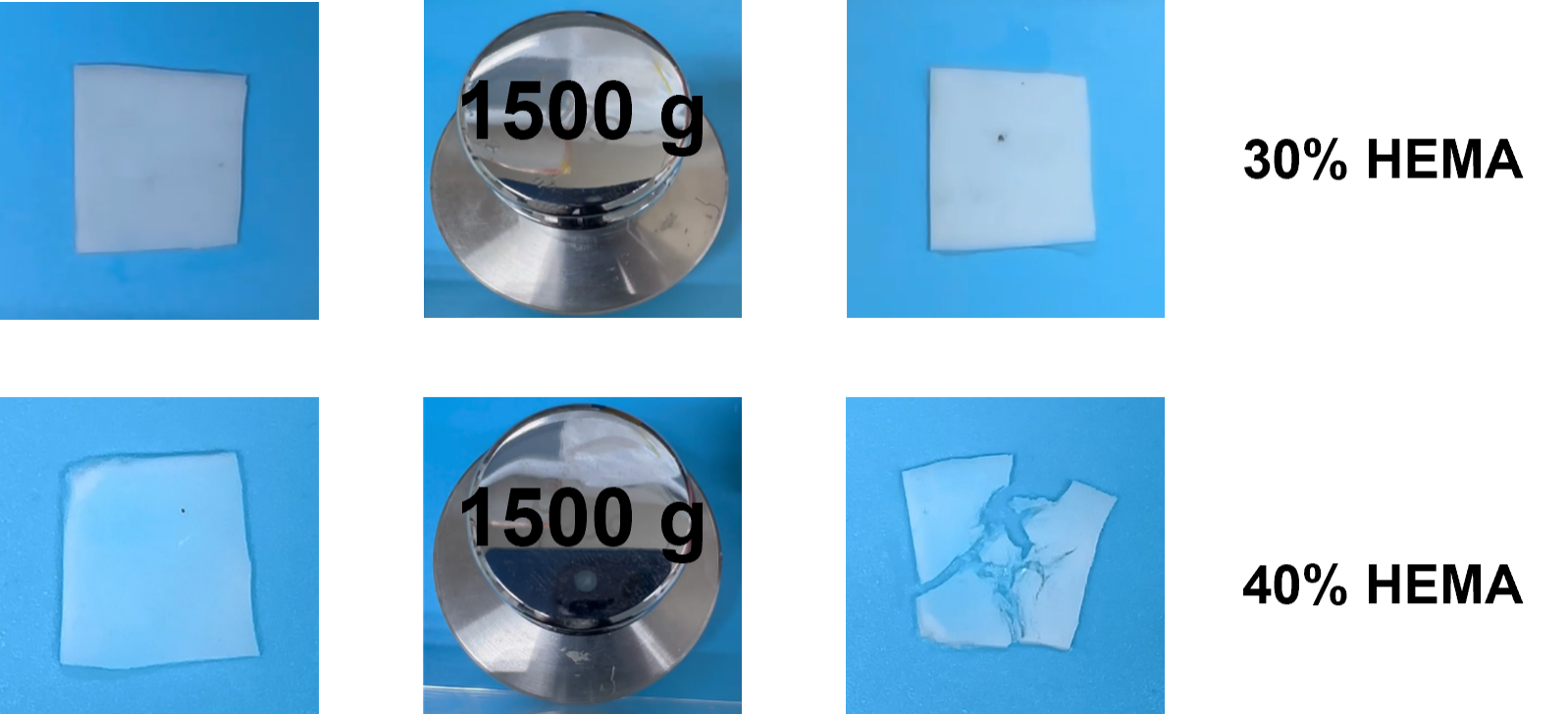


**Fig. S7.** Images of the toughness test of the actuating layer with 30% and 40% HEMA content.


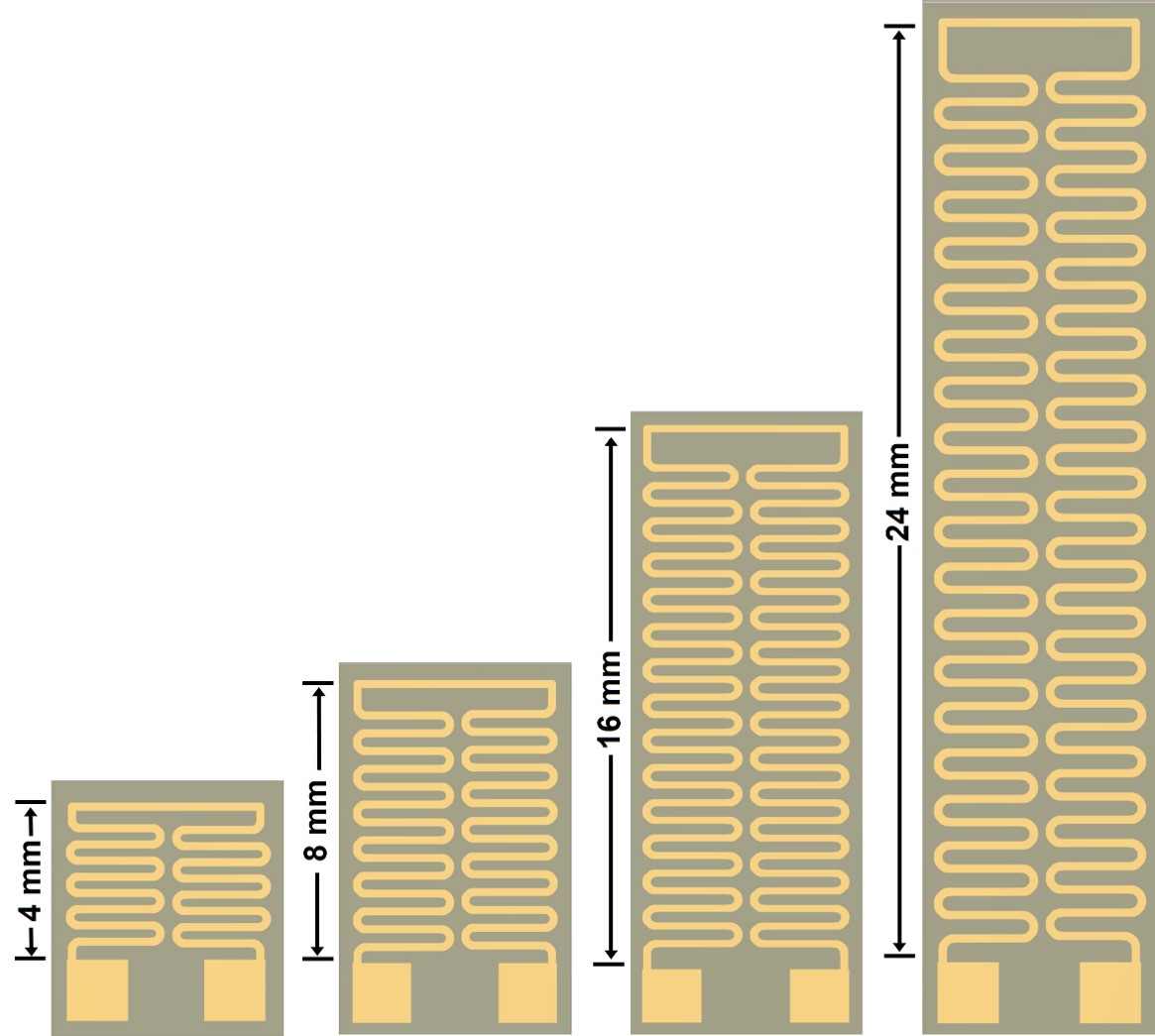


**Fig. S8.** Images of 4 mm, 8 mm, 16 mm, and 24 mm length heaters.


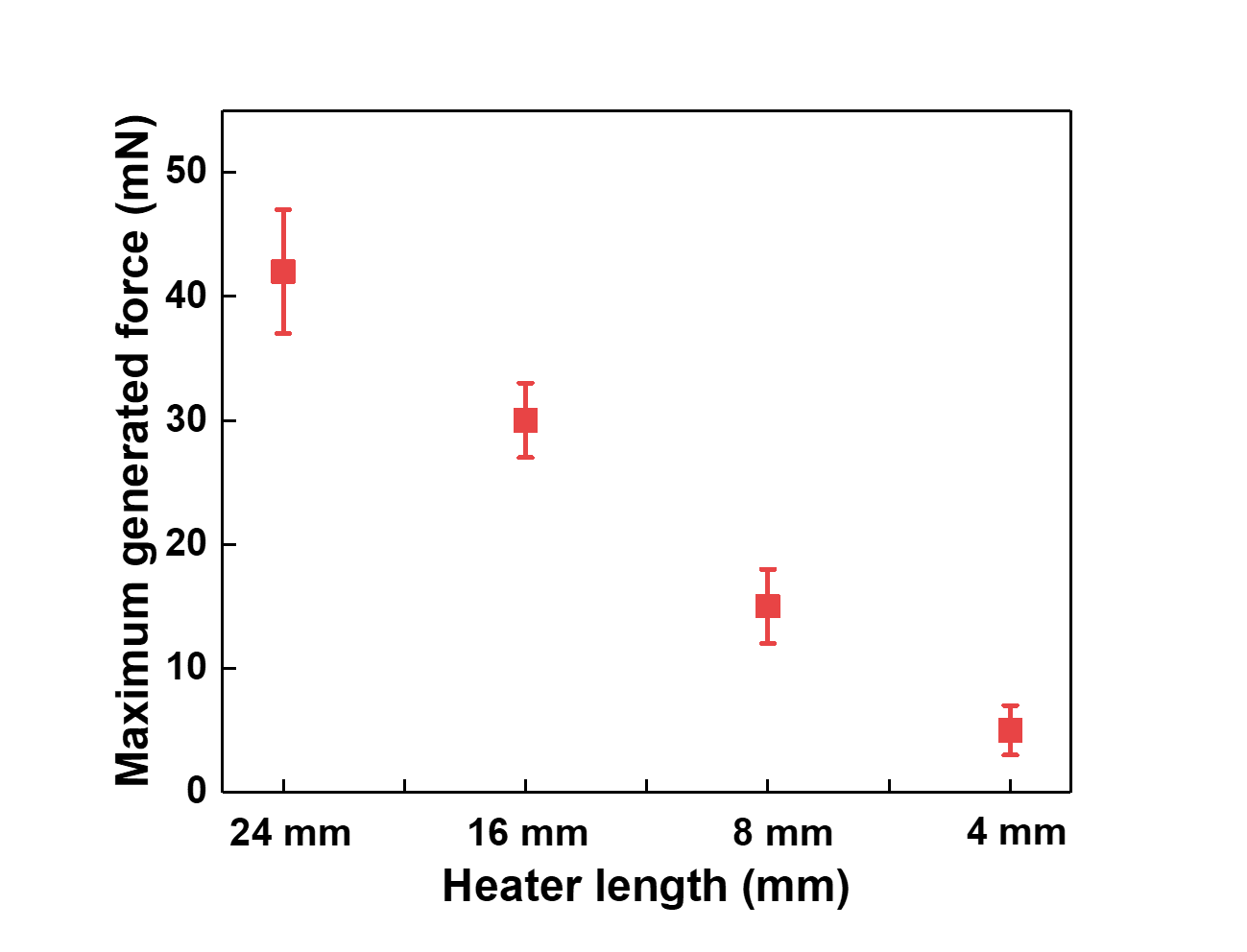


**Fig. S9.** The generated force of the hydrogel muscle with different heater lengths.


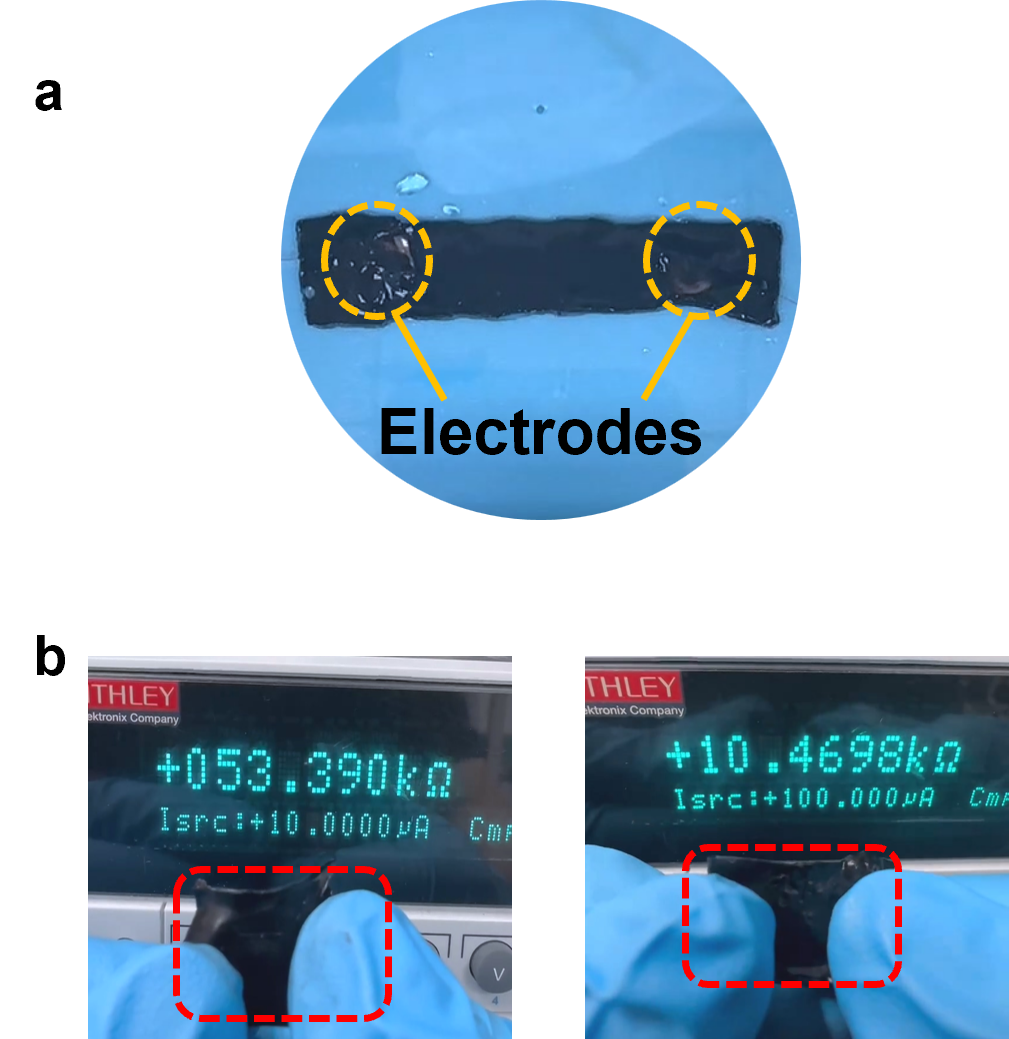


**Fig. S10. a** Image of the sensing layer with inserted electrodes. **b** Images of the sensing layer resistance without (left) and with (right) liquid metal.


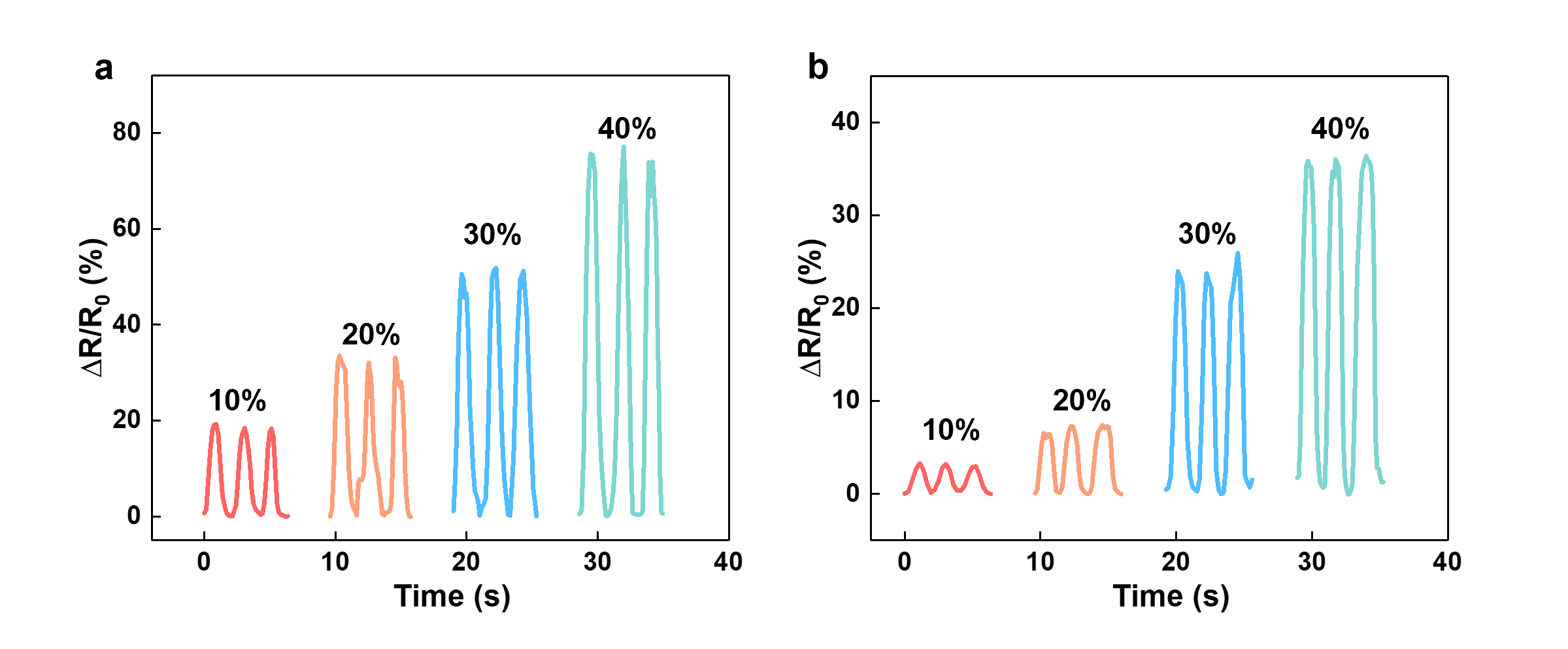


**Fig. S11.** Resistance changes of the sensing layer **a** with LM and **b** without LM at 10% to 40% strain.


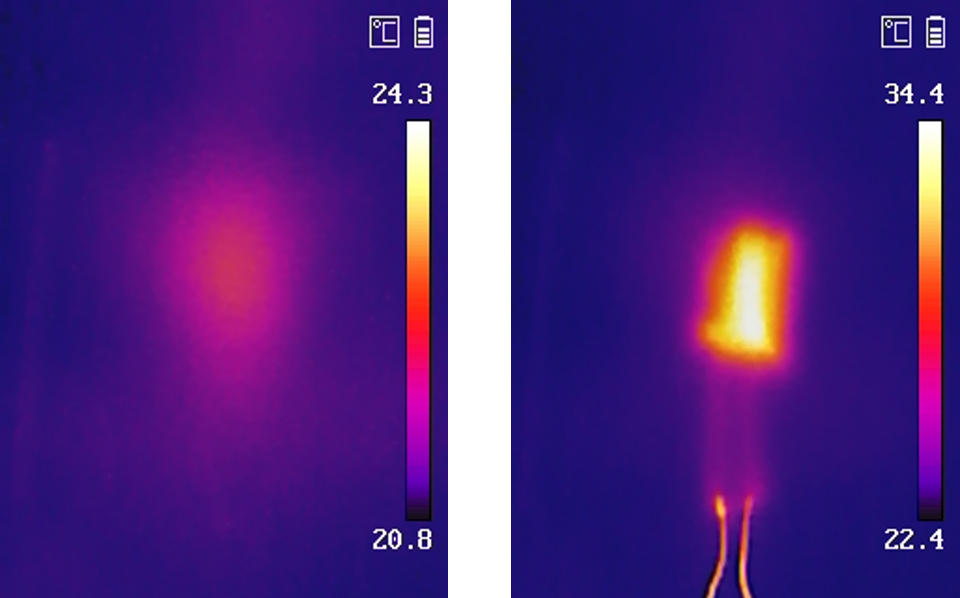


**Fig. S12.** Thermal image of the heater in the initial state and after power on for 8 s.


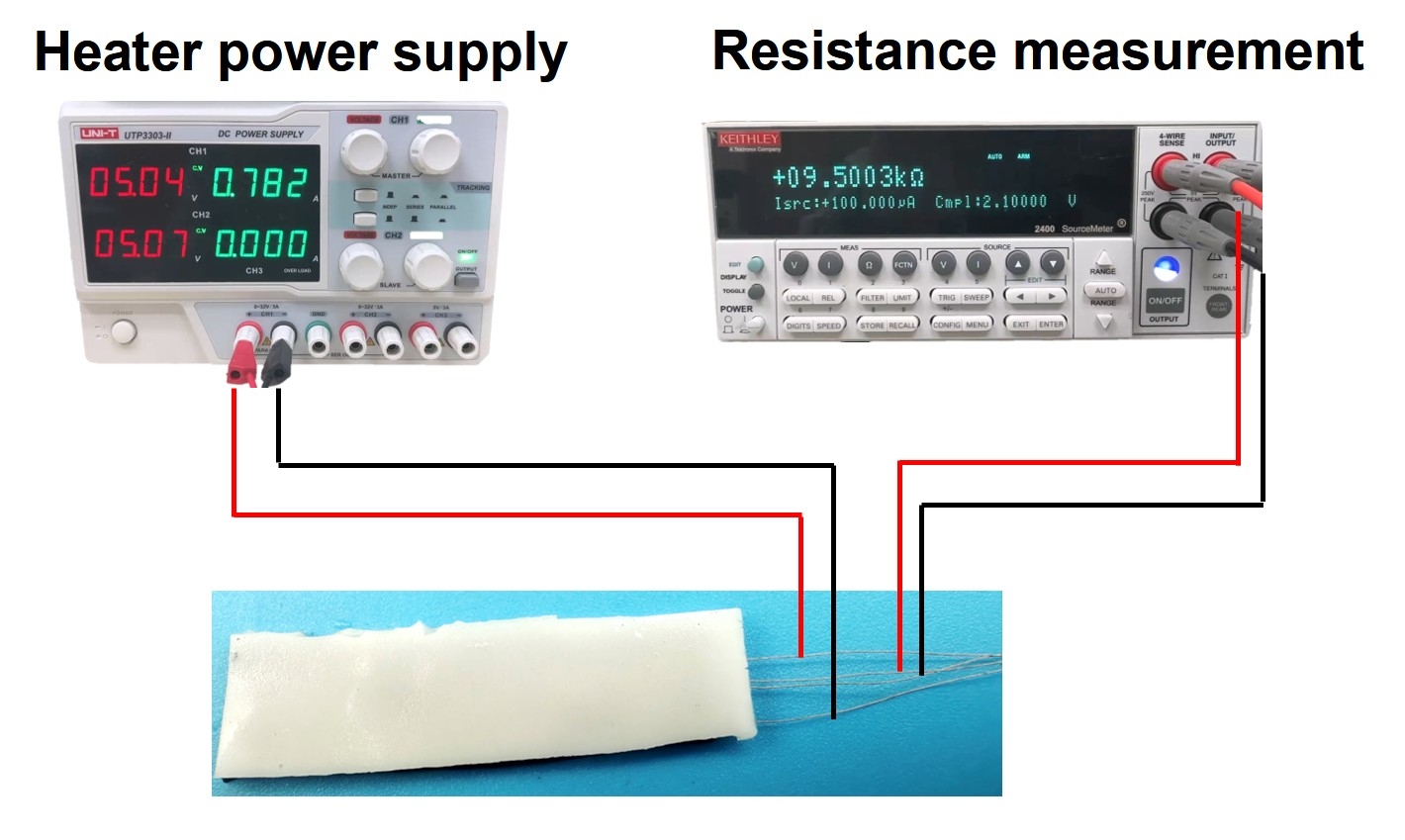


**Fig. S13.** Actuation and measurement system of demonstration in **Figure 4.**


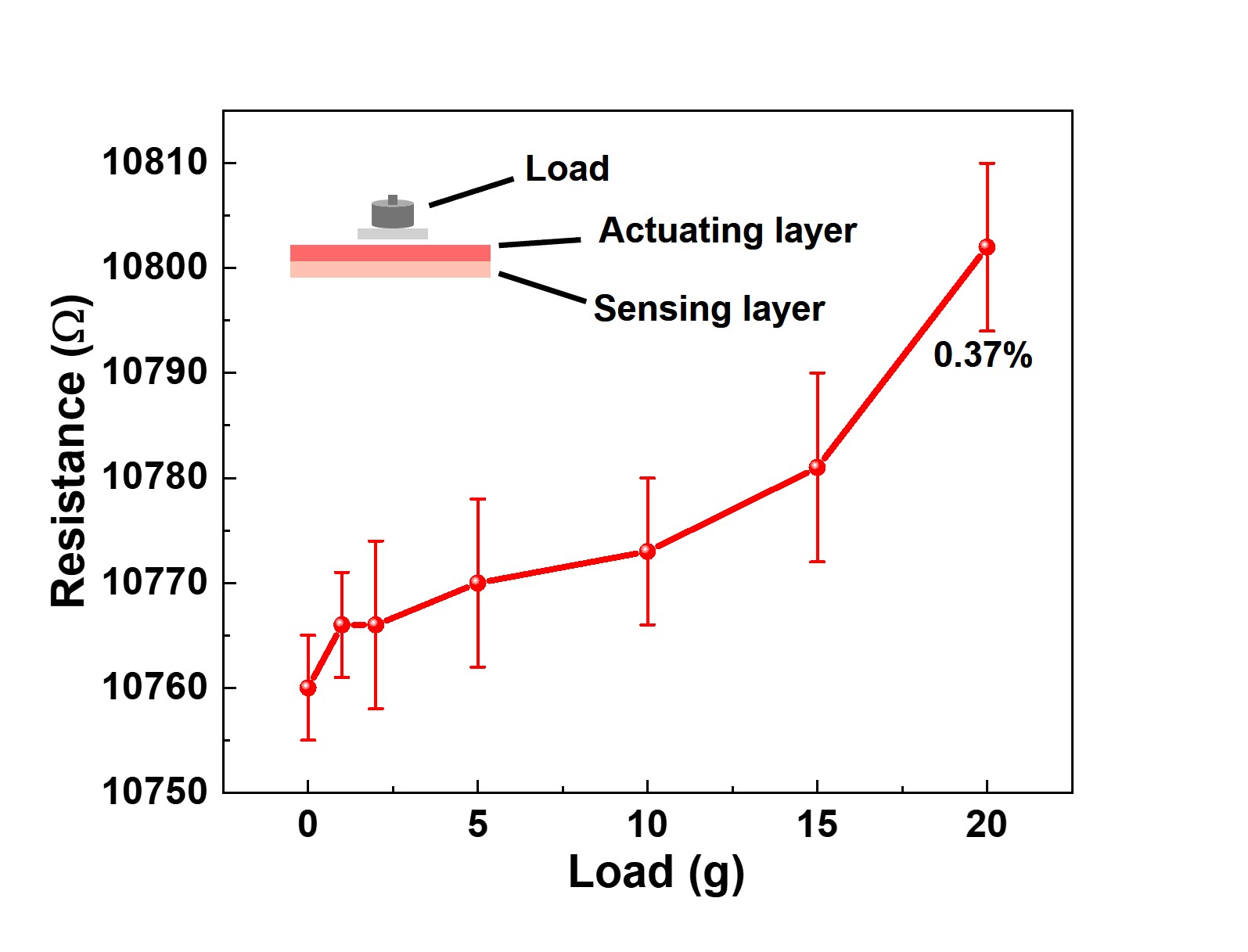


**Fig. S14.** Resistance changes of the sensing layer under different load weights.


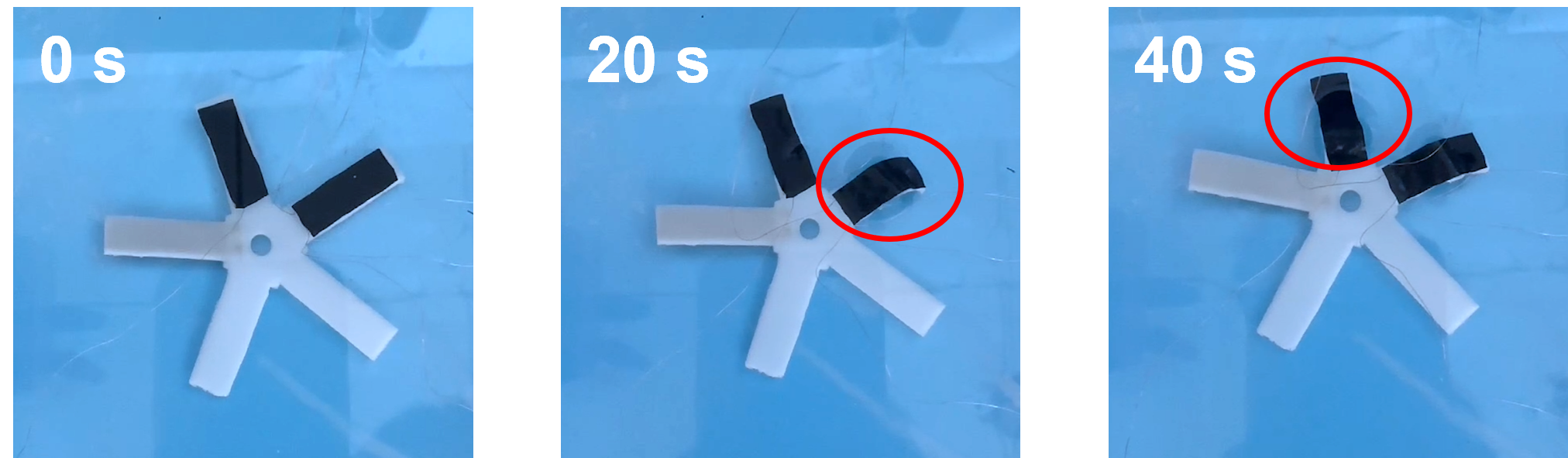


**Fig. S15.** Images show the moving of a starfish shaped robot.


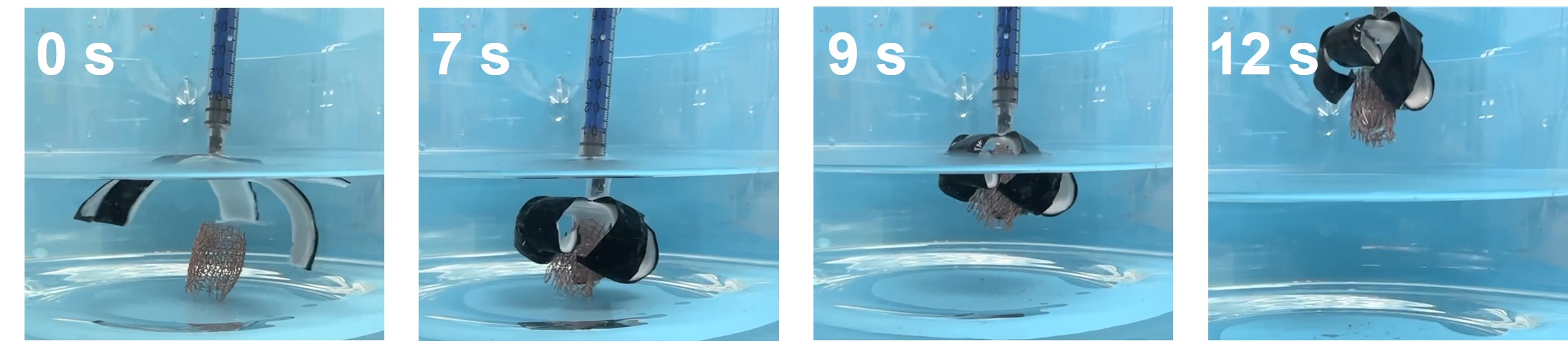


**Fig. S16.** Demonstration of the hydrogel muscle gripper in 35℃ water.


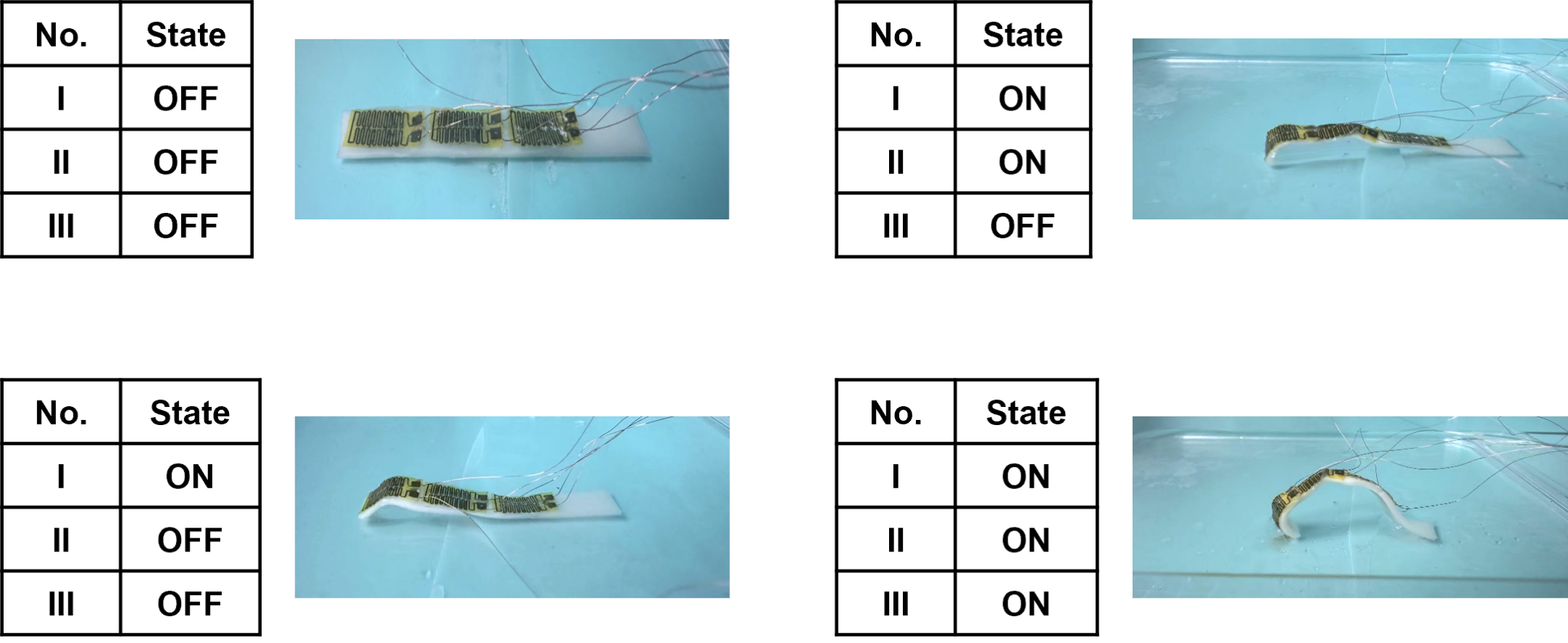


**Fig. 17** Images of the actuating layer integrated with 3 heaters to realize the programmable deformation.

**
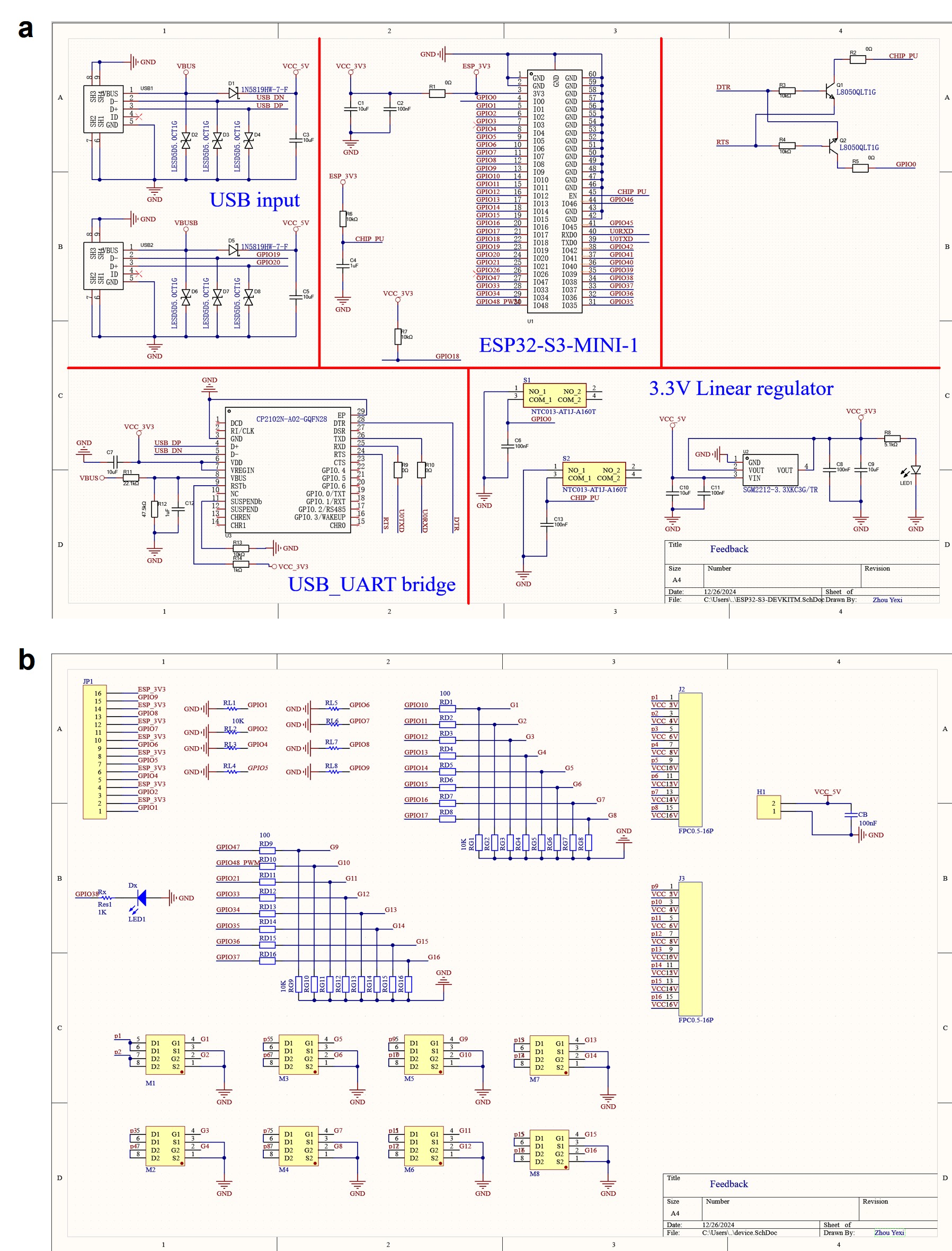
**

**Fig. S18.** Circuit schematic diagram of the close-loop control demonstration.

**
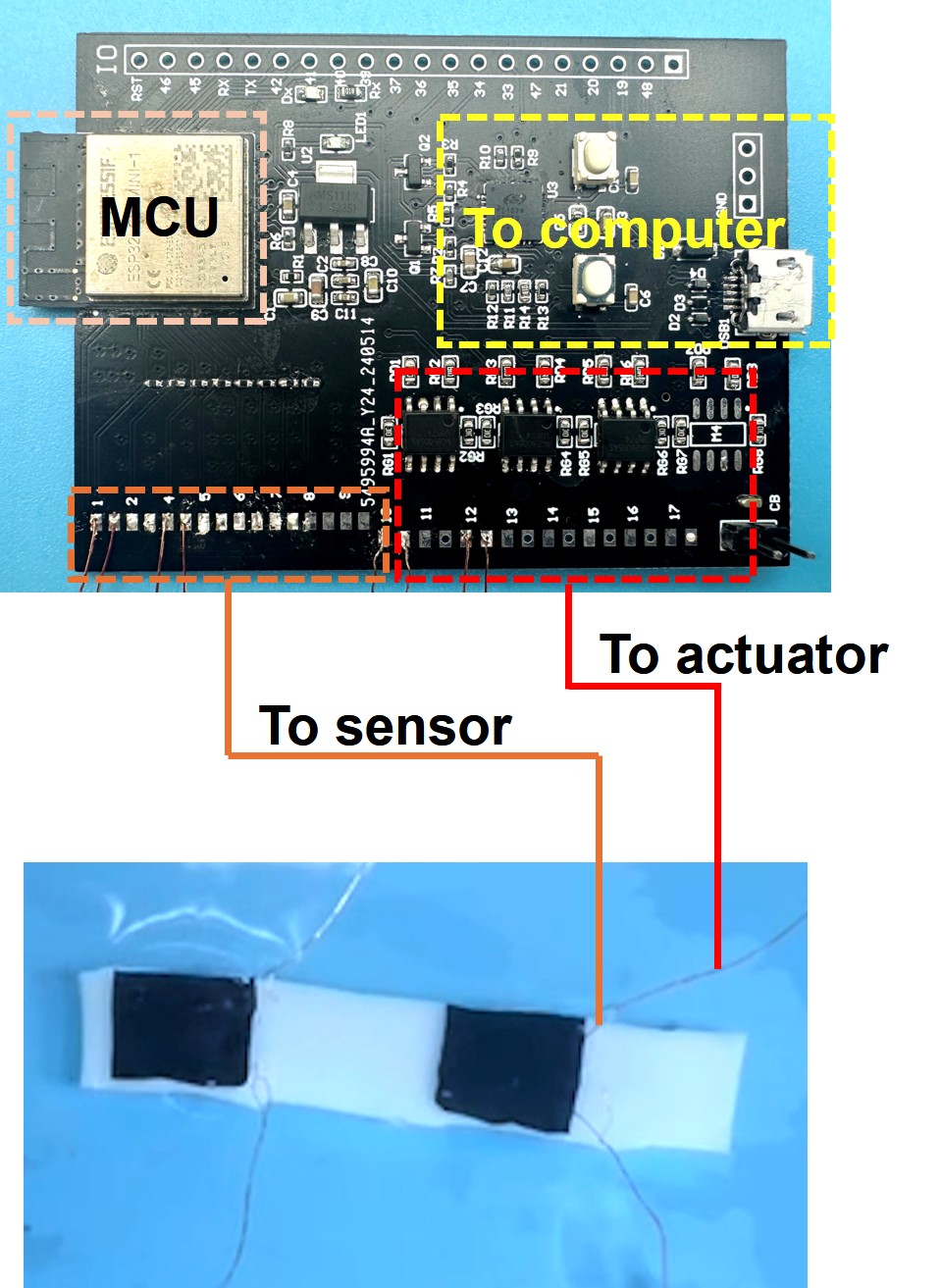
**

**Fig. S19.** Image of the close-loop control system and the connection method.

**Supplementary Video captions**

**Video S1:** Deswelling and swelling process of the 30% HEMA content actuator layer hydrogel.

**Video S2:** Bending and recovery process of 1 mm actuator/0.5 mm sensor layer hydrogel composite.

**Video S3:** Bending process under different voltages.

**Video S4:** Lifting different weight loads.

**Video S5:** Motion state of the starfish shaped robot.

**Video S6:** Demonstration of the hydrogel muscle gripper in 35℃ water.

**Video S7:** Demonstration of the self-crawling robot.
